# Are phase resetting curves tunable?

**DOI:** 10.1186/1471-2202-15-S1-P75

**Published:** 2014-07-21

**Authors:** Sorinel A Oprisan, Davy Vanderweyen, Derek Tuck

**Affiliations:** 1 Department of Physics and Astronomy, College of Charleston, Charleston, SC 29424, USA

## 

**Figure 1:**
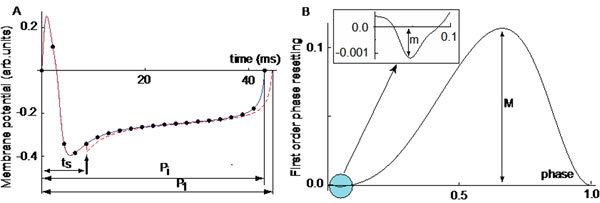
(A) Intrinsic oscillations (continuous line) and transiently perturbed period (dashed line). (B) A Type I PRC is bimodal with one large lobe of amplitude M and the other with a disproportionately smaller amplitude m.

Neurons are excitable cells capable of sustaining high amplitude membrane potential oscillations called action potential (AP). There are two classes of repetitively firing neurons: Class 1 characterized by a continuously and arbitrarily low tunable firing frequency, or Class 2 characterized by a minimum firing frequency threshold [1]. The ionic channels and the morphology of the cells determine the characteristics of repetitive firing, such as the intrinsic period P_i_, the firing threshold, or the duration of the AP. For the study of phase locked modes and synchrony of neural populations a phenomenological understanding of neural activity suffices. In such cases, the phase response curve (PRC) rather than a detailed biophysical model of the neuron provides accurate and quick results. A PRC tabulates the transient changes in the firing period of a neuron due to an external perturbation, usually a brief current or synaptic conductance pulse [2,3]. The most significant effect of a perturbation occurs during the cycle that contains the perturbation and is quantified by the first order PRC, i.e. F_1_(φ) = P_1_/P_i_ - 1, where P_1_ is the transiently modified duration of the current cycle due to a perturbation applied at the stimulus time t_s_ or phase φ = t_s_/P_i_ (Figure 1A). There are two types of PRCs: Type II PRC that has an almost sinusoidal shape and comparable sizes of the positive and negative lobes, and Type I PRC that has a disproportionate ratio of the two lobes (see Figure 1B). When Type I neurons are part of neural network they can predominantly speed up (or only slow down) their rhythm, whereas Type II neurons synchronize more readily due to their ability to both slow down and speed up. While it is clear that every PRCs are bimodal, the purpose of this study is to investigate what parameters determine their shape. We found that the phase space bifurcation structure determines not only the PRC’s type but also how easily the shape of the PRC can be tuned such that a “typical” Type I PRC can be smoothly converted into a Type II PRC. We found that nonlinear oscillators characterized both by saddle-node of invariant circle (SNIC) and Andronov-Hopf (HB) bifurcations, such as Morris-Lecar (ML) model neuron, the PRC can be smoothly adjusted from a pure Type I to a Type II. For example, by slowly increasing a bias current, ML model reaches a SNIC bifurcation and produces a typical Type I PRC approximately captured by the generic infinitesimal PRC iPRC_1_(φ) = 1 - cos(φ). Further increasing the bias current brings the model closer to a HB that produces a typical Type II infinitesimal PRC iPRC_2_(φ) = sin(φ + α), with α a constant phase. For any intermediate value of the bias current the actual shape of the infinitesimal PRC is a linear combination of iPRC_1_ and iPRC_2_.
